# Estimation of Bond Strength and Effective Bond Length for the Double Strap Joint between Carbon Fiber Reinforced Polymer (CFRP) Plate and Corroded Steel Plate

**DOI:** 10.3390/polym14153069

**Published:** 2022-07-29

**Authors:** Anbang Li, Hao Wang, Han Li, Deliang Kong, Shanhua Xu

**Affiliations:** 1 School of Civil Engineering, Xi’an University of Architecture & Technology, Xi’an 710055, China; lianbang@xauat.edu.cn (A.L.); lihan@live.xauat.edu.cn (H.L.); kongdel228@163.com (D.K.); 2 State Key Laboratory of Green Building in Western China, Xi’an 710055, China; 3 Key Laboratory of Structural Engineering and Earthquake Resistance, Ministry of Education, Xi’an 710055, China; 4 MCC Group, Central Research Institute of Building and Construction Co., Ltd., Beijing 100088, China

**Keywords:** carbon fiber reinforced polymer, corroded steel plate, bond strength, effective bond length, estimation model

## Abstract

In this paper, we examine the development of the estimation models of bond strength and effective bond length for a double strap joint between carbon fiber reinforced polymer (CFRP) plate and corroded steel plate. The experimental study on the bond behavior between CFRP plate and corroded steel plate is summarized first and the analytical interfacial bond–slip model for CFRP plate externally bonded to corroded steel plate is proposed. Based on the theoretical stress analysis for the CFRP plate–corroded steel plate double-lap joint, the piecewise expressions of the interfacial shear stress and the normal peel stress of the interface between CFRP plate and corroded steel plate were established. The estimation models of the bond strength and the effective bond length for the double strap joint between the CFRP plate and the corroded steel plate were consequently developed on the basis of interfacial stress distribution equations and the stress boundary conditions. The comparison between the predicted and experimental results indicated that the proposed models could be adopted to predict the bond strength and effective bond length for the CFRP plates externally bonded to corroded steel substrates with reasonable accuracy. The proposed estimation models are expected to provide meaningful references and essential data for the reliable application of CFRP strengthening system to the performance improvement of corroded steel structures.

## 1. Introduction

Corrosion is an almost unavoidable durability problem for many steel structures which have been exposed to long-time corrosive environments such as industry and ocean atmosphere [1]. To prolong the service life of corroded steel structures, compared with the possible long-term traffic interruption, production line shutdown and other economic consequences caused by the direct demolition or replacement of rusted components, the reinforcement and repair of corroded steel structures is usually a more economical and effective method. Carbon fiber reinforced polymer (CFRP) materials possess the characteristics of high strength/weight ratio as well as excellent fatigue and corrosion resistance, and the strengthening system with CFRP bonded to a steel substrate has been proved to be more efficient by minimizing the additional permanent load, eliminating stress concentration, no residual stress or new structural damage, and higher durability than the traditional repair and reinforcement methods such as welding, bolting or riveting [2,3]. Li et al [4] tested the fatigue behavior of corroded steel plates strengthened with CFRP plates; they demonstrated that the fatigue life of the patched specimen which presented the most significant strengthening effectiveness showed a fatigue life extension of more than 85.3 times with respect to the unpatched corroded steel plate and approximately twice more than the uncorroded steel plate; the strengthening system with external bond CFRPs presented a good engineering application prospect in improving the performance of corroded steel structures. The bonding interface between CFRP and corroded steel plate is the weakest position of the whole reinforcement system, and thus the knowledge of the bond properties for CFRP plates externally bonded to corroded steel substrates such as the stress distribution, bond strength and effective bond length, are the basis for the engineering application of CFRP in strengthening corroded steel structures.

A great deal of researches have been concerned with the bond properties between CFRP material and steel plate. In terms of experimental study, Jiao and Zhao [5], Fawzia et al. [6] and Al-Mosawe et al. [7] have tested the bond characteristics between steel plate and CFRP sheet (or plate) with different modulus of elasticity. The effect of adhesive types which usually classified as liner and non-liner adhesives, on the bond behavior between the externally bonded CFRP and steel substrate were investigated by Yu et al. [8] and Peng [9]. The influence of adhesive thickness on the bond properties between CFRP and steel plate were studied by Xia and Teng [10], Wang et al. [11] and Li et al. [12]. The effects of surface preparation methods which included solvent cleaning, gridding with sandpaper and grit blasting, etc., on the bonding performance of the interface between CFRP and steel plate were compared by Kim et al. [13] and Fernando et al. [14]. Several different bond stress-slip models for the CFRP-steel interface also have been developed by Xia and Teng [10], Dehghani et al. [15], Fawzia et al. [16], He and Xian [17], Wang and Wu [11], Pang et al. [18]. In terms of theoretical analysis, the earliest model for calculating the ultimate bearing capacity of CFRP-steel double shear laps joints might be proposed by Hart-Smith [19] based on the study on the bond behavior between glued metallic materials. The basic one-dimensional linear-elastic analysis for tapered double-doubler joints and double-sided reinforcements were conducted by Albat and Romilly [20], and the direct one-dimensional linearelastic solution of the adhesive shear stress distribution and the adherend normal stress distribution for the interface between CFRP and steel plate was presented. The numerical analysis on the stress transfer and fracture propagation process for four different kinds of adhesive joints in FRP-strengthened steel structure were carried out by Wu et al. [21], expressions for the maximum transferable load and interfacial shear stress distribution were also established by introducing two types of nonlinear interface bonding constitutive models to describe pre and post-interfacial microdebonding behavior between CFRP and steel plate. Deng et al. [22] presented an analytical solution to calculate the stresses in the reinforced beam under mechanical as well as thermal loads. Peng [9] presented the interfacial stress analysis for CFRP double-sided reinforced steel plate and flexural steel beam external bonded with FRP on the tensile flange, and the models for estimating bond strength and effective bond length of these two kinds of bonding interface were also established. A calculation model for ultimate bearing capacity and effective bond length of single lapped interface between CFRP plate and steel plate corresponding to linear elastic adhesive was proposed by Xia and Teng [10] based on the first ever developed bond-slip model and fracture mechanics method. Fernando [23], further proposed the calculation model of ultimate bearing capacity and effective bond length of CFRP-steel single lapped interface corresponding to nonlinear adhesive. The fracture mechanics analysis on the bonding interface of double-lap joint between CFRP plate and steel plate were carried out by Bocciarelli [24], and the analytical solutions of the interfacial shear stress and normal stress distribution, and the calculation model of interface bond strength based on maximum strain energy release rate theory were also proposed. Deng and Huang [25] presented an analytical solution to calculate the interfacial stresses in the beams strengthened with a CFRP plate under mechanical loads, thermal loads and prestressing actions. In general, the bond properties between CFRP material and steel plate have been systematically investigated through experimental study and theoretical analysis.

However, what should be pointed out is that almost all the aforementioned studies were focused on the bond behavior between CFRP materials and the intact steel substrates, i.e., the uncorroded steel surface, only a very small amount of experimental work has been involved with the bond properties for CFRP material externally bonded to corroded steel substrates [12,26,27]. The limited experimental data have shown that the increase in surface area, surface free energy and surface roughness caused by corrosion would took positive effect on the ultimate bearing capacity of the bonding interface, and longer CFRP plates may be required to ensure the effectiveness of load transformation when the CFRP strengthening system is adopted for corroded steel structures [12]. To the authors’ knowledge, however, there have been no studies executed to quantitatively estimate the ultimate load (bond strength) and effective bond length for CFRP plates externally bonded to corroded steel substrates, of which the computational model is the foundation of engineering applications for CFRP materials in strengthening corroded steel structures.

The main purpose of this study is to establish the calculation models to estimate the ultimate load (bond strength) and effective bond length of the bonding interface between CFRP and corroded steel plate. The recent test results conducted by the authors are summarized first and the analytical interfacial bond–slip model for CFRP plate externally bonded to corroded steel plate is proposed. Based on the present test data and proposed bond–slip model, the theoretical stress analysis of the bonding interface between CFRP plate and corroded steel plate was carried out, and the distribution equations of interfacial shear stress and normal peel stress were established. Finally, the estimation models of the ultimate load (bond strength) and effective bonding length for the double-lap joint between CFRP plate and corroded steel plate were developed combined with the stress boundary conditions, and the comparison between the predicted values and experimental results was made to verify the accuracy of the estimation models.

## 2. Summary of a New Experimental Study

To investigate the bond behavior between CFRP plate and corroded steel plate, the authors of this paper recently conducted a series of double-lap joint tensile tests on the bond behavior of CFRP plates externally bonded to corroded steel substrates [12]. The bond characteristics, including failure modes, ultimate load, CFRP strain and interfacial shear stress distribution, and effective bond length of the double-lap joint specimens were tested and analyzed together with the effect of corrosion duration on the surface topography and roughness, and the surface free energy of the corroded steel plates. The main factors to be considered included the corrosion duration, the bond length and the adhesive thickness. The steel substrates used in this study were the corroded steel plates cut from the flanges of the hot rolled Q235 H 350 × 175 × 7 × 11 beams with corrosion durations *C*_d_ of 0, 3, 4, 6, 8 and 12 months, respectively. The unidirectional CFRP plate with a width of 50 mm and a thickness of 1.4 mm was adopted. Thixotropic and solventless two-part epoxy Sikadur-30CN was used to bond the CFRP plates. The main mechanical properties for all the materials obtained from coupon tensile tests are listed in Table 1. Thirty-four double-lap joints were tested to study the bond behavior between CFRP plates and corroded steel plates, as listed in Table 2. The specimens’ geometry was as follows: the double-lap joint was fabricated with two CFRP plates bonded to two corroded steel plates, the dimensions of the CFRP plates and the corroded steel plates were (2*L* + 20) mm × 35 mm × 1.4 mm and 200 mm × 35 mm × *t_c_* mm (length × width × thickness), respectively. Where *L* was the intended bond length of CFRP plate for the testing side of specimen, and the bond length of the anchorage side of the specimen was larger than *L* by 20 mm to make sure the failure always happened on the testing side. *t_c_* was the thickness of the corroded steel plates, *t_c_* = 10.75*(1 − *ξ*), where 10.75 was the thickness of the uncorroded steel plate, *ξ* was weight loss rate of the corroded steel plate. Five kinds of bond length *L* which included 30, 50, 80, 120 and 150 mm, were prepared for the double-lap joints. The intended adhesive thickness varied from 0.5 mm to 1.0 mm, 1.5 mm and 2.0 mm, and the average values of the measured thickness of both side of adhesive layers (*t*_ave_) are listed in Table 2. Before fabricating the specimens, the corrosion products of the steel surfaces were removed carefully using an electric steel wire brush, and then the dust and greasy dirt of the corroded steel surfaces were cleaned using anhydrous alcohol. More details of the specimens can be found in the authors’ recent study [12]. All specimens were loaded under tension with a force-displacement hybrid control program, i.e., a load-controlled step was first executed until the tension load reached the threshold value of 3 kN and then a displacement controlled step with a loading rate of 0.5 mm/min was carried out until specimen failure.

Four kinds of failure modes which included steel/adhesive interfacial debonding, cohesive failure, CFRP/adhesive interfacial debonding, and CFRP delamination were observed in the experimental study. Results showed that the failure mode of the double-lap joints mainly depended on the adhesive thickness rather than the corrosion duration, the failure modes of the double-lap joints with the same corrosion duration changed from the combination of steel/adhesive interfacial failure and CFRP/adhesive interfacial failure to the combination of CFRP/adhesive interfacial failure and CFRP delamination accompanied with the intended adhesive thickness increasing from 0.5 mm to 2.0 mm. The results also showed that corrosion was found to have a positive effect on the ultimate load (bond strength) for the double-lap joints with the same failure mode of steel/adhesive interfacial failure; moreover, with regard to the specimens of the same corrosion duration, the ultimate load increased at first and decreased afterwards with the intended adhesive thickness increasing from 0.5 to 1.0, 1.5 and 2.0 mm. The effective bond lengths of the double-lap joints with a corrosion duration of 0–12 months and a measured adhesive thickness of 0.21–2.1 mm were approximately 67.75–107.5 mm. As for the specimens of the same corrosion duration, the effective bond length progressively increased with increasing adhesive thickness, in addition, regarding the specimens with similar measured adhesive thickness, the effective bond length of the corroded specimens was obviously larger than that of the uncorroded specimens. The main test results of all the specimens are listed in Table 2.

The CFRP strain distribution was recorded by a series of strain gauges pasted on the CFRP plates, and the interfacial bond stress and relative slip for CFRP plates externally bonded to the corroded steel substrates could be deduced based on the force balance condition of the micro elements of the CFRP plate.

(1) $$\tau_{i∼i+1}=\frac{\left(\sigma_{c,i}-\sigma_{c,i+1}\right)t_{c}}{L_{i∼i+1}}=\frac{\left(\varepsilon_{c,i}-\varepsilon_{c,i+1}\right)E_{c}t_{c}}{L_{i∼i+1}}$$

(2) $$s_{i∼i+1}=\left(\frac{1}{2}+\frac{E_{c}t_{c}}{E_{s}t_{s}}\right)\left(\varepsilon_{c,i}+\varepsilon_{c,i+1}\right)L_{i∼i+1}-\frac{2\varepsilon_{c,1}E_{c}t_{c}}{E_{s}t_{s}}L_{i∼i+1}$$

where $\tau_{i,i+1}$ is the average interfacial shear stress of the bonding interface between the corresponding positions of the adjacent strain gauges *i* and *i* + 1, $s_{i∼i+1}$ is the relative slip of the bonding interface between the corresponding positions of the adjacent strain gauges *i* and *I* + 1, $L_{i∼i+1}$ is the distance between the adjacent strain gauges *i* and *i* + 1, $\sigma_{c,i}$ and $\sigma_{c,i+1}$ are the tensile stresses of CFRP plate at the corresponding positions of strain gauges *i* and *I* + 1, respectively, $\varepsilon_{c,i}$ and $\varepsilon_{c,i+1}$ are the tensile strains of the CFRP plate at the corresponding positions of strain gauges *i* and *i* + 1, $E_{c}$ and $t_{c}$ are the elastic modulus and thickness of CFRP plates, respectively, $E_{s}$ and $t_{s}$ are the elastic modulus and average thickness of corroded steel plates, respectively.

(3) $$t_{s}=\left(1-\xi \right)t_{0}$$

where $t_{0}$ is the thickness of the uncorroded steel plate, *ξ* is the weight loss rate of the corroded steel plate. 

**Figure 1:**
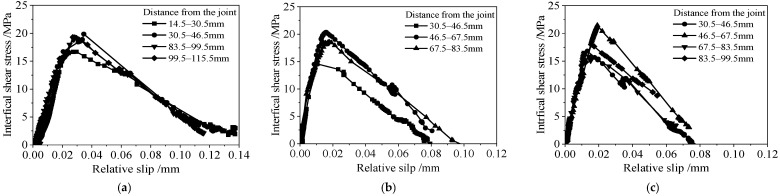
Typical bond stress-slip curves of interface between CFRP plate and corroded steel plate: (**a**) specimen C3-B5-T2, (**b**) specimen C6-B5-T1 and (**c**) specimen C8-B5-T1.

Figure 1 presents the typical bond–slip relationships for the specimens with different adhesive thicknesses and corrosion durations. As shown in Figure 1, the bond–slip curves calculated from CFRP strain data at different locations were similar in shape and had typical two-stage characteristics; that is, with the increase in relative slip, the interfacial shear stress increased at first and decreased afterward. However, the specific characteristic values of the curves which included the curve convexity of the rising section, peak interfacial shear stress, slip at peak interfacial shear stress, and maximum slip at interface failure, were significantly affected by the adhesive thickness and corrosion duration of the steel plate. 

**Figure 2:**
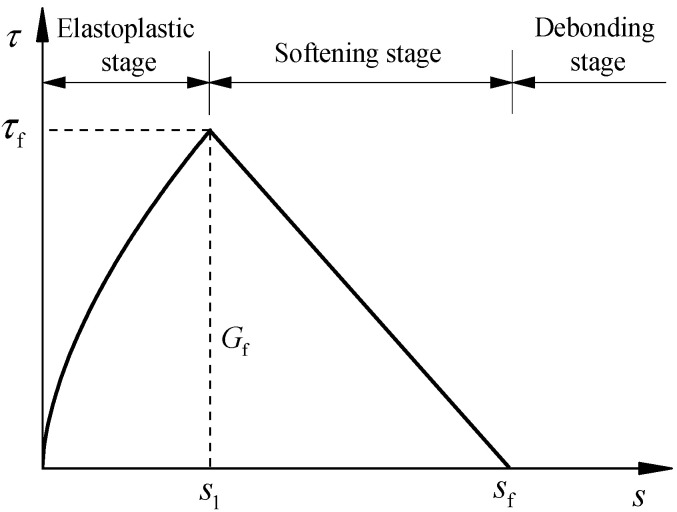
Bond–slip model for CFRP plate and corroded steel plate.

**Figure 3:**
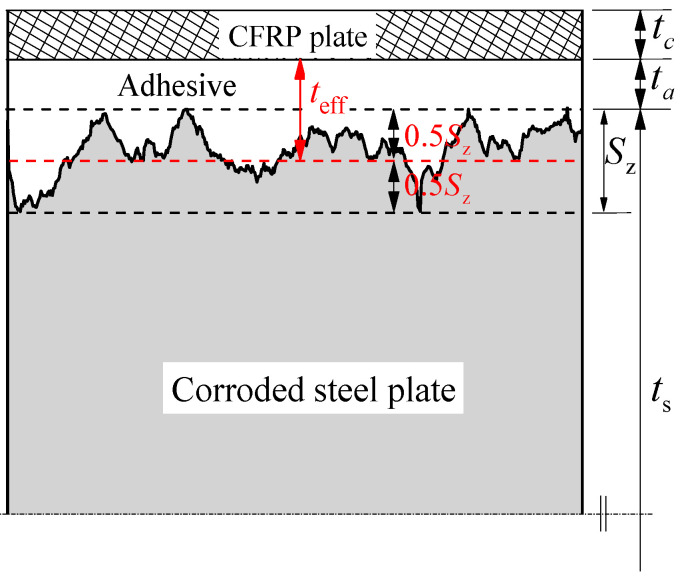
Schematic diagram of the cross section for the bonding interface between CFRP plate and corroded steel plate.

Herein, the accurate bond stress-slip models with which the effect of adhesive thickness and corrosion could be taken into consideration were proposed, see Figure 2. As Figure 2 illustrates, the analytical bond stress-slip model was formulated by the following three stages, i.e., the elastoplastic stage, softening stage and complete failure stage, and the general expressions could be expressed as following:

(4) $$\tau =\left\{ \begin{array}{l} \tau_{f}{\left(\frac{s}{s_{1}}\right)}^{\alpha }\;,s\leq s_{1}\\ \tau_{f}\frac{s_{f}-s}{s_{f}-s_{1}}\;,s_{1}<s\leq s_{f}\\ 0\;,s>s_{f} \end{array}\right.$$

where *τ* is bond stress, *s* is relative slip, is the peak interfacial shear stress, $s_{1}$ is the slip corresponding to the peak interfacial shear stress, *α* is the fitting parameter of the curve rising section, $s_{f}$ is the maximum slip at interface failure. The above characteristic parameters were calibrated according to the fitting results of test data, and more details about the calculation process can be found in the authors’ recent study [28]:

(5) $$\tau_{f}=0.5f_{t,a}$$

(6) $$\alpha =\tanh(1.1t_{\mathrm{eff}})$$

(7) $$s_{1}=0.0059t_{\mathrm{eff}}+0.0174$$

(8) $$s_{f}=\frac{2G_{f}}{\tau_{f}}-\frac{1-\alpha }{1+\alpha }s_{1}$$

(9) $$G_{f}=-0.1827t_{\mathrm{eff}}^{2}+0.6494t_{\mathrm{eff}}+0.5919$$

where $f_{t,a}$ is the tensile strength of the adhesive, $G_{f}$ is the interfacial fracture energy, $t_{\mathrm{eff}}$ is the effective adhesive thickness, which is defined as the sum of the construction adhesive thickness and the additional adhesive thickness caused by the corroded rough surface, as shown in Figure 3.

(10) $$t_{\mathrm{eff}}=t_{a}+0.5S_{z}$$

where *S*_z_ is the maximum height of the corroded steel surface, it reflects the two extreme distributions of the surface roughness [29], and can be calculated by applying the following equation:

(11) $$S_{z}=\underset{(x,y)\in A}{\max}\left[z(x,y)\right]-\underset{(x,y)\in A}{\min}\left[z(x,y)\right]$$

where *A* is the nominal area of the scanning surface, *z*(*x, y*) is the measured height of the scale-limited surface at position (*x, y*). The values of *S*_z_ for all the corroded steel plates with different corrosion durations are listed in Table 2; more details of the calculation process and surface scanning can be found in the authors’ recent study [12]. 

## 3. Several Simplified Assumptions

In the following sections, based on the analytical interfacial bond–slip model proposed previously, the theoretical stress analysis of the bonding interface between CFRP plate and corroded steel plate was carried out first, and then the distribution equations of interfacial shear stress and normal peel stress of the interface between CFRP plate and corroded steel plate were established. Finally, the estimation models of the ultimate load (bond strength) and effective bonding length for the bonding interface between CFRP plate and corroded steel plate were developed combined with the stress boundary condition of the double-lap joint. To properly simplify the stress analysis process, the following four simplified assumptions were proposed:

1. The width of the CFRP plate, corroded steel plate and adhesive layer for the double-lap joint are equal, and the thickness of these three parts remain unchanged along the loading direction;
2. The tensile stress of the CFRP plate and corroded steel plate are evenly distributed along their thickness directions;
3. The adhesive layer only transmits interfacial shear stress and normal peel stress, and does not bear horizontal tensile stress;
4. The pouring and curing temperature of the specimen is consistent with the loading ambient temperature, and the influence of thermal stress is ignored.

**Figure 4:**
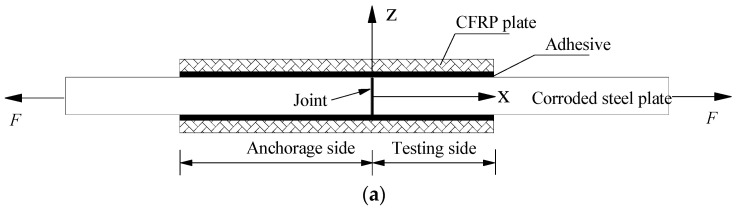

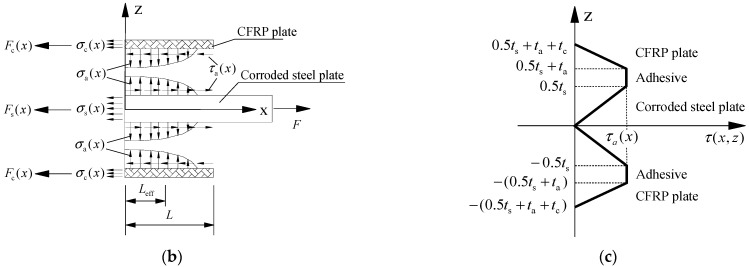
Schematic diagram of stress analysis for the interface between CFRP plate and corroded steel plate: (**a**) composition of the double-lap joint, (**b**) interfacial stress distribution along the bond length, and (**c**) interfacial shear-stress distribution along the specimen thickness direction.

According to the above simplified assumptions, Figure 4a–c illustrates the schematic composition diagram of the double-lap joint, the interfacial stress distribution along the CFRP bond length, and the interfacial shear stress distribution along the specimen thickness direction, respectively. In Figure 4, all symbols with subscripts “a” representing the adhesive layer, “s” representing the steel plate and “C” representing the CFRP plate.

## 4. Theoretical Stress Analysis

**Figure 5:**
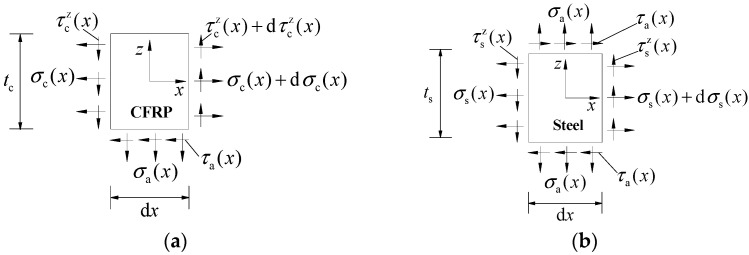
Stress analysis diagram of (**a**) CFRP plate microsegment and (**b**) steel plate microsegment.

In conjunction with the chosen coordinate system shown in Figure 4, Figure 5a,b presents the stress analysis diagram of CFRP plate microsegment and corroded steel plate microsegment, respectively. According to the force balance conditions, the equilibrium differential equations of CFRP plate microsegment and steel plate microsegment can be expressed as:

(12) $$d\sigma_{c}(x)=\frac{\tau_{a}(x)dx}{t_{c}}$$

(13) $$d\tau_{c}^{z}(x)=\frac{\sigma_{a}(x)dx}{t_{c}}$$

(14) $$\tau_{c}^{z}(x)=\frac{\tau_{a}(x)}{2}$$

(15) $$d\sigma_{s}(x)=\frac{-2\tau_{a}(x)dx}{t_{s}}$$

where and $t_{s}$ are the thickness of the CFRP plate and corroded steel plate, respectively. $\sigma_{c}(x)$ and $\sigma_{s}(x)$ are the tensile stress of the CFRP plate and corroded steel plate, respectively. $\tau_{a}(x)$ and $\sigma_{a}(x)$ are the horizontal shear stress and normal peel stress of the adhesive layer, respectively. $\tau_{c}^{z}(x)$ is the shear stress along the thickness direction in the CFRP plate microsegment.

According to the geometric relationship between deformation and displacement, the geometric equations of the CFRP plate microsegment and corroded steel plate microsegment can be expressed as:

(16) $$\varepsilon_{c}(x)=\frac{\partial u_{c}(x)}{\partial x}=\frac{\sigma_{c}(x)}{E_{c}}$$

(17) $$\varepsilon_{s}(x)=\frac{\partial u_{s}(x)}{\partial x}=\frac{\sigma_{s}(x)}{E_{s}}$$

where $\varepsilon_{c}(x)$ and $\varepsilon_{s}(x)$ are the axial tensile strain of the CFRP plate and steel plate, respectively, and their values are assumed to be uniformly distributed along the section according to the aforementioned second simplified assumption. $u_{c}(x)$ and $u_{s}(x)$ are the axial tensile deformation of the CFRP plate and steel plate, respectively. $E_{c}$ and $E_{s}$ are the elastic modulus of the CFRP plate and steel plate, respectively.

According to Figure 4b, the stress boundary conditions are established as follows:

(18) $$F_{c}(x)=t_{c}b_{c}\sigma_{c}(x)$$

(19) $$F_{s}(x)=t_{s}b_{s}\sigma_{s}(x)$$

(20) $$F=2F_{c}(x)+F_{s}(x)$$

where $F_{c}(x)$ and $F_{s}(x)$ are the tensile force of the CFRP plate and corroded steel plate of unit width, respectively. $b_{c}$ an $b_{s}$ are the width of the CFRP plate and corroded steel plate, respectively.

Considering that the shear modulus of both the corroded steel plate and the CFRP plate present a much larger value than that of the adhesive, the relative slip of the bonding interface can be calculated by ignoring the relative slip caused by the shear deformation of the corroded steel plate and the CFRP plate, and thus the relative slip value s(x) of the bonding interface at position *x* can be expressed as:

(21) $$s(x)=u_{c}(x)-u_{s}(x)$$

At the same time, the relative slip value s(x) meets the interfacial bond–slip model established previously, that is

(22) $$\tau_{a}(x)=f(s(x))=\left\{ \begin{array}{l} \tau_{f}{\left(\frac{s}{s_{1}}\right)}^{\alpha },\;s(x)\leq s_{1}\\ \tau_{f}\frac{s_{f}-s}{s_{f}-s_{1}}\;,\;s_{1}<s(x)\leq s_{f}\\ 0\;,\;s(x)>s_{f} \end{array}\right.$$

Taking the derivative on both sides of Equation (21) and substituting it into Equations (16) and (17) yields

(23) $$\frac{ds}{dx}=\frac{du_{c}(x)}{dx}-\frac{du_{s}(x)}{dx}=\frac{\sigma_{c}(x)}{E_{c}}-\frac{\sigma_{s}(x)}{E_{s}}$$

According to Equations (18)–(20), it can be obtained that

(24) $$\sigma_{s}(x)=\frac{F-2t_{c}b_{c}\sigma_{c}(x)}{t_{s}b_{s}}$$

Substituting Equation (24) into Equation (23) yields

(25) $$\frac{ds}{dx}=\sigma_{c}(x)\left(\frac{1}{E_{c}}+\frac{2t_{c}b_{c}}{t_{s}b_{s}E_{s}}\right)-\frac{F}{t_{s}b_{s}E_{s}}$$

Rewriting Equation (25) yields

(26) $$\sigma_{c}(x)=\left(\frac{ds}{dx}+\frac{F}{t_{s}b_{s}E_{s}}\right)\frac{1}{\left(\frac{1}{E_{c}}+\frac{2t_{c}b_{c}}{t_{s}b_{s}E_{s}}\right)}$$

Defining $\lambda^{2}$

(27) $$\lambda^{2}=\frac{\tau_{f}^{2}}{2G_{f}}\left(\frac{1}{t_{c}E_{c}}+\frac{2b_{c}}{t_{s}b_{s}E_{s}}\right)$$

where $G_{f}$ and $\tau_{f}$ are the interfacial fracture energy and the peak interfacial shear stress, which can be calculated from Equations (9) and (5), respectively. Rewriting Equation (26) yields

(28) $$\sigma_{c}(x)=\frac{\tau_{f}^{2}}{2G_{f}t_{c}\lambda^{2}}\left(\frac{ds}{dx}+\frac{F}{t_{s}b_{s}E_{s}}\right)$$

Substituting Equations (22) and (28) into Equation (12) yields

(29) $$\frac{d^{2}s}{dx^{2}}-\frac{2G_{f}\lambda^{2}}{\tau_{f}^{2}}f(s(x))=0$$

Substituting Equation (22) into Equation (29) yields the following piecewise functions,

If $s(x)\leq s_{1}$,

(30) $$\frac{d^{2}s}{dx^{2}}-\frac{2G_{f}\lambda^{2}}{\tau_{f}^{}}{\left(\frac{s}{s_{1}}\right)}^{\alpha }=0$$

If $s_{1}<s(x)\leq s_{f}$,

(31) $$\frac{d^{2}s}{dx^{2}}+\frac{2G_{f}\lambda^{2}}{\tau_{f}^{}}\frac{s}{s_{f}-s_{1}}=\frac{2G_{f}\lambda^{2}}{\tau_{f}^{}}\frac{s_{f}}{s_{f}-s_{1}}$$

Defining $\lambda_{1}{}^{2}$ and $\lambda_{2}{}^{2}$

(32) $$\lambda_{1}^{2}=\lambda^{2}\frac{2G_{f}}{\tau_{f}^{}s_{1}^{\alpha }}$$

(33) $$\lambda_{2}^{2}=\lambda^{2}\frac{2G_{f}}{\tau_{f}^{}\left(s_{f}-s_{1}\right)}$$

Rewriting Equations (30) and (31) yields

(34) $$\frac{d^{2}s}{dx^{2}}-\lambda_{1}^{2}s^{\alpha }=0$$

(35) $$\frac{d^{2}s}{dx^{2}}+\lambda_{2}^{2}s=\lambda_{2}^{2}s_{f}$$

To solve Equations (34) and (35), it was noted that the parameter α in Equation (34), only reflects the outward protruding shape characteristic of the rising section of the bond–slip curve, and its value is approximately equal to 1, see Equation (6). To simplify the solving process of the differential equation, the value of parameter α is temporarily taken as 1.0, and thus, the general solution of Equations (34) and (35) can be expressed as:

If $s(x)\leq s_{1}$,

(36) $$s(x)=A\cosh\left(\lambda_{1}\left(L-x\right)\right)+B\sinh\left(\lambda_{1}\left(L-x\right)\right)$$

Substituting Equation (36) into Equations (22) and (28) yields

(37) $$\tau_{a}(x)=\frac{\tau_{f}}{s_{1}}\left(A\cosh\left(\lambda_{1}\left(L-x\right)\right)+B\sinh\left(\lambda_{1}\left(L-x\right)\right)\right)$$

(38) $$\sigma_{c}(x)=\frac{\tau_{f}^{2}}{2G_{f}t_{c}\lambda^{2}}\left(-A\lambda_{1}\sinh\left(\lambda_{1}\left(L-x\right)\right)-B\lambda_{1}\cosh\left(\lambda_{1}\left(L-x\right)\right)+\frac{F}{t_{s}b_{s}E_{s}}\right)$$

If $s_{1}<s(x)\leq s_{f}$, the bond–slip relationship has entered the softening stage (see Figure 2), which means that there is already a softening section on the bonding interface. Herein, assuming that the length of the softening section is *a*, then the general solution of Equation (35) can be expressed as:

(39) $$s(x)=C\sin\left(\lambda_{2}\left(a-x\right)\right)+D\cos\left(\lambda_{2}\left(a-x\right)\right)+s_{f}$$

Accordingly,

(40) $$\tau_{a}(x)=\frac{-\tau_{f}}{s_{f}-s_{1}}\left(C\sin\left(\lambda_{2}\left(a-x\right)\right)+D\cos\left(\lambda_{2}\left(a-x\right)\right)\right)$$

(41) $$\sigma_{c}(x)=\frac{\tau_{f}^{2}}{2G_{f}t_{c}\lambda^{2}}\left(-C\lambda_{2}\cos\left(\lambda_{2}\left(a-x\right)\right)+D\lambda_{2}\sin\left(\lambda_{2}\left(a-x\right)\right)+\frac{F}{t_{s}b_{s}E_{s}}\right)$$

The parameters *A*, *B*, *C* and *D* in the above general solution can be solved according to the stress boundary conditions of the double-lap joint. As shown in Figure 4b, at the position of *x* = 0, Equation (38) yields

(42) $$\sigma_{c}(x=0)=\frac{F}{2b_{c}t_{c}}$$

Substituting Equation (42) into Equation (38) yields

(43) $$B=\frac{F}{t_{s}b_{s}E_{s}\lambda_{1}}$$

Since that the bond–slip relationship is continuous at $s=s_{1}$, the following relationships are satisfied for Equations (37) and (40),

(44) $$\tau_{a}(x=a)=\tau_{f}$$

(45) $$s\left(x=a\right)=s_{1}$$

Substituting Equation (44) into Equation (37) yields

(46) $$A=\frac{s_{1}-\frac{F}{t_{s}b_{s}E_{s}\lambda_{1}}\sinh\left(\lambda_{1}\left(L-a\right)\right)}{\cosh\left(\lambda_{1}\left(L-a\right)\right)}$$

Substituting Equation (45) into Equation (39) yields

(47) $$D=s_{1}-s_{f}$$

Since the bond–slip relationship is continuous at $s=s_{1}$, Equations (38) and (41) are compatible when *x* = *a*,

(48) $$C=\frac{\lambda_{1}s_{1}\tanh\left(\lambda_{1}\left(L-a\right)\right)}{\lambda_{2}}\;+\frac{F}{\lambda_{2}t_{s}b_{s}E_{s}}\left(\cosh\left(\lambda_{1}\left(L-a\right)\right)-\sinh\left(\lambda_{1}\left(L-a\right)\right)\tanh\left(\lambda_{1}\left(L-a\right)\right)\right)$$

The relative slip s(x) and interfacial shear stress $\tau_{a}(x)$ of the CFRP plates externally bonded to corroded steel substrates, and the tensile stress $\sigma_{c}(x)$ of the CFRP plate can be obtained by substituting the values of *A*, *B*, *C* and *D* into Equations (36)–(41). The normal peel stress of the bonding interface $\sigma_{a}\left(x\right)$ and the tensile stress of corroded steel plate $\sigma_{s}\left(x\right)$ can be obtained according to Equations (13) and (14) and Equations (18)–(20), respectively. The piecewise expressions of the above physical quantities are as follows:

When $s(x)\leq s_{1}$, a≤x≤L,

(49) $$s(x)=\frac{s_{1}-\frac{F}{t_{s}b_{s}E_{s}\lambda_{1}}\sinh\left(\lambda_{1}\left(L-a\right)\right)}{\cosh\left(\lambda_{1}\left(L-a\right)\right)}\cosh\left(\lambda_{1}\left(L-x\right)\right)\;+\frac{F\sinh\left(\lambda_{1}\left(L-x\right)\right)}{t_{s}b_{s}E_{s}\lambda_{1}}$$

(50) $$\tau_{a}(x)=\frac{\tau_{f}-\frac{F\tau_{f}}{s_{1}t_{s}b_{s}E_{s}\lambda_{1}}\sinh\left(\lambda_{1}\left(L-a\right)\right)}{\cosh\left(\lambda_{1}\left(L-a\right)\right)}\cosh\left(\lambda_{1}\left(L-x\right)\right)+\frac{F\tau_{f}\sinh\left(\lambda_{1}\left(L-x\right)\right)}{s_{1}t_{s}b_{s}E_{s}\lambda_{1}}$$

(51) $$\sigma_{a}\left(x\right)=\frac{\frac{Ft_{c}\tau_{f}}{s_{1}t_{s}b_{s}E_{s}}\sinh\left(\lambda_{1}\left(L-a\right)\right)-\lambda_{1}\tau_{f}t_{c}}{2\cosh\left(\lambda_{1}\left(L-a\right)\right)}\sinh\left(\lambda_{1}\left(L-x\right)\right)-\frac{Ft_{c}\tau_{f}\cosh\left(\lambda_{1}\left(L-x\right)\right)}{2s_{1}t_{s}b_{s}E_{s}\lambda_{1}}$$

(52) $$\sigma_{c}(x)=\frac{\sinh\left(\lambda_{1}\left(L-x\right)\right)}{\cosh\left(\lambda_{1}\left(L-a\right)\right)}\frac{FE_{c}\sinh\left(\lambda_{1}\left(L-a\right)\right)-s_{1}\lambda_{1}t_{s}b_{s}E_{s}E_{c}}{t_{s}b_{s}E_{s}+2b_{c}t_{c}E_{c}}+\frac{E_{c}F\left(1-\cosh\left(\lambda_{1}\left(L-x\right)\right)\right)}{t_{s}b_{s}E_{s}+2b_{c}t_{c}E_{c}}$$

(53) $$\begin{array}{l} \sigma_{s}(x)=\frac{F}{t_{s}b_{s}}-\frac{\frac{2t_{c}b_{c}}{t_{s}b_{s}}E_{c}F\left(1-\cosh\left(\lambda_{1}\left(L-x\right)\right)\right)}{t_{s}b_{s}E_{s}+2b_{c}t_{c}E_{c}}\\ \;\;\;-\frac{\frac{2t_{c}b_{c}}{t_{s}b_{s}}\sinh\left(\lambda_{1}\left(L-x\right)\right)}{\cosh\left(\lambda_{1}\left(L-a\right)\right)}\frac{FE_{c}\sinh\left(\lambda_{1}\left(L-a\right)\right)-s_{1}\lambda_{1}t_{s}b_{s}E_{s}E_{c}}{t_{s}b_{s}E_{s}+2b_{c}t_{c}E_{c}} \end{array}$$

When $s_{1}<s(x)\leq s_{f}$, 0≤x≤a,

(54) $$s(x)=\frac{\lambda_{1}s_{1}\tanh\left(\lambda_{1}\left(L-a\right)\right)\sin\left(\lambda_{2}\left(a-x\right)\right)}{\lambda_{2}}+\frac{F\sin\left(\lambda_{2}\left(a-x\right)\right)}{\lambda_{2}t_{s}b_{s}E_{s}\cosh\left(\lambda_{1}\left(L-a\right)\right)}+\left(s_{1}-s_{f}\right)\cos\left(\lambda_{2}\left(a-x\right)\right)+s_{f}$$

(55) $$\tau_{a}(x)=\frac{-\tau_{f}\lambda_{1}s_{1}\tanh\left(\lambda_{1}\left(L-a\right)\right)\sin\left(\lambda_{2}\left(a-x\right)\right)}{\left(s_{f}-s_{1}\right)\lambda_{2}}\;-\frac{\tau_{f}F\sin\left(\lambda_{2}\left(a-x\right)\right)}{\left(s_{f}-s_{1}\right)\lambda_{2}t_{s}b_{s}E_{s}\cosh\left(\lambda_{1}\left(L-a\right)\right)}+\tau_{f}\cos\left(\lambda_{2}\left(a-x\right)\right)$$

(56) $$\sigma_{a}(x)=\frac{\tau_{f}\lambda_{1}s_{1}t_{c}\tanh\left(\lambda_{1}\left(L-a\right)\right)\cos\left(\lambda_{2}\left(a-x\right)\right)}{2\left(s_{f}-s_{1}\right)}\;+\frac{t_{c}\tau_{f}F\cos\left(\lambda_{2}\left(a-x\right)\right)}{2\left(s_{f}-s_{1}\right)t_{s}b_{s}E_{s}\cosh\left(\lambda_{1}\left(L-a\right)\right)}+\frac{t_{c}\tau_{f}\lambda_{2}\sin\left(\lambda_{2}\left(a-x\right)\right)}{2}$$

(57) $$\sigma_{c}(x)=-\left(\frac{\lambda_{1}s_{1}t_{s}b_{s}E_{s}\sinh\left(\lambda_{1}\left(L-a\right)\right)+F}{\cosh\left(\lambda_{1}\left(L-a\right)\right)}\right)\;\frac{E_{c}\cos\left(\lambda_{2}\left(a-x\right)\right)}{t_{s}b_{s}E_{s}+2b_{c}t_{c}E_{c}}+\frac{FE_{c}+t_{s}b_{s}E_{s}E_{c}\left(s_{1}-s_{f}\right)\lambda_{2}\sin\left(\lambda_{2}\left(a-x\right)\right)}{t_{s}b_{s}E_{s}+2b_{c}t_{c}E_{c}}$$

(58) $$\begin{array}{l} \sigma_{s}(x)=\frac{F}{b_{s}t_{s}}+\left(\frac{\lambda_{1}s_{1}\tanh\left(\lambda_{1}\left(L-a\right)\right)}{\lambda_{2}}\;+\frac{F}{\lambda_{2}t_{s}b_{s}E_{s}\cosh\left(\lambda_{1}\left(L-a\right)\right)}\right)\frac{2b_{c}t_{c}E_{s}E_{c}\lambda_{2}\cos\left(\lambda_{2}\left(a-x\right)\right)}{t_{s}b_{s}E_{s}+2b_{c}t_{c}E_{c}}\\ \;\;\;-\frac{\frac{2b_{c}t_{c}}{b_{s}t_{s}}FE_{c}+2b_{c}t_{c}E_{s}E_{c}\left(s_{1}-s_{f}\right)\lambda_{2}\sin\left(\lambda_{2}\left(a-x\right)\right)}{t_{s}b_{s}E_{s}+2b_{c}t_{c}E_{c}} \end{array}$$

## 5. Bond Strength and Effective Bond Length

According to Figure 4b, the following stress boundary condition is obtained:

(59) $$\sigma_{c}\left(x=0\right)=\frac{F}{2b_{c}t_{c}}$$

Substitute Equation (59) into Equation (57) yields

(60) $$F=\frac{\left(s_{1}-s_{f}\right)\lambda_{2}\sin\left(\lambda_{2}a\right)-\lambda_{1}s_{1}\tanh\left(\lambda_{1}\left(L-a\right)\right)\cos\left(\lambda_{2}a\right)\;}{\left(\frac{1}{2b_{c}t_{c}E_{c}}+\frac{\cos\left(\lambda_{2}a\right)}{t_{s}b_{s}E_{s}\cosh\left(\lambda_{1}\left(L-a\right)\right)}\right)}$$

It was found from Equation (60) that the ultimate load (bond strength) is a function of the CFRP bond length *L* and the softening section length *a*, nevertheless, from the perspective of experimental study and theoretical analysis, when the predetermined bond length of the CFRP plate exceeded the effective bond length ($L_{\mathrm{eff}}$), the increase in the ultimate load of the bonding interface could almost be ignored [12]. Therefore, the following two boundary conditions were obtained: ① *F* is convergent with the increase in *L*; ② the maximum value of *F* is reached when *dF/da* = 0. By observing Equation (60), it was found that when the value of bond length *L* far exceeds the effective bonding length, i.e., the bond length *L* takes a large value, the following relationship is obviously true:

(61) $$\underset{L\to +\infty }{\lim}\cosh\left(\lambda_{1}\left(L-a\right)\right)=+\infty$$

(62) $$\underset{L\to +\infty }{\lim}\tanh\left(\lambda_{1}\left(L-a\right)\right)=1$$

Substituting Equations (61) and (62) into Equation (60) yields

(63) $$F_{u}=\underset{L\to +\varpi }{\lim}F=2b_{c}t_{c}E_{c}\left[\left(s_{1}-s_{f}\right)\lambda_{2}\sin\left(\lambda_{2}a\right)-\lambda_{1}s_{1}\cos\left(\lambda_{2}a\right)\right]\;$$

where $F_{u}$ is the ultimate load (bond strength) of the double-lap joint.

The following equations can be derived from $dF_{u}/da=0$:

(64) $$\tan\left(\lambda_{2}a\right)=\frac{\lambda_{2}^{}}{\lambda_{1}s_{1}}\left(s_{f}-s_{1}\right)=\sqrt{\frac{s_{f}-s_{1}}{s_{1}}}$$

(65) $$a=\frac{1}{\lambda_{2}}\arctan\sqrt{\frac{s_{f}-s_{1}}{s_{1}}}=\frac{\arctan\sqrt{\frac{s_{f}-s_{1}}{s_{1}}}}{\sqrt{\frac{\tau_{f}^{}}{s_{f}-s_{1}}\left(\frac{1}{t_{c}E_{c}}+\frac{2b_{c}}{t_{s}b_{s}E_{s}}\right)}}$$

Substituting Equation (64) into Equation (63), the following expression for the bond strength is proposed:


(66) $$F_{u}=2b_{c}t_{c}E_{c}\sqrt{\tau_{f}^{}s_{f}\left(\frac{1}{t_{c}E_{c}}+\frac{2b_{c}}{t_{s}b_{s}E_{s}}\right)}\;=2b_{c}t_{c}E_{c}\sqrt{2G_{f}\left(\frac{1}{t_{c}E_{c}}+\frac{2b_{c}}{t_{s}b_{s}E_{s}}\right)}$$

Substituting Equation (65) into Equation (60) yields

(67) $$F=\frac{\left(s_{f}-s_{1}+s_{1}\tanh\left(\lambda_{1}\left(L-a\right)\right)\right)\sqrt{\frac{\tau_{f}^{}}{s_{f}}\left(\frac{1}{t_{c}E_{c}}+\frac{2b_{c}}{t_{s}b_{s}E_{s}}\right)}}{\left(\frac{1}{2b_{c}t_{c}E_{c}}+\frac{\sqrt{\frac{s_{1}}{s_{f}}}}{t_{s}b_{s}E_{s}\cosh\left(\lambda_{1}\left(L-a\right)\right)}\right)}$$

It can be seen from Equation (67) that *F* increases with the increase ofbond length *L*, and finally converges to the maximum value of $F_{u}$. Herein, the effective bond length ($L_{\mathrm{eff}}$) is defined as the corresponding bond length of the CFRP plate when the bearing capacity reaches 99.99% of its ultimate load (bond strength) ($F_{u}$); consequently, it can be obtained from Equations (66) and (67) that

(68) $$\left(s_{f}-s_{1}+s_{1}\tanh\left(\lambda_{1}\left(L_{\mathrm{eff}}-a\right)\right)\right)\approx 0.9999s_{f}$$

Substituting Equation (65) into Equation (68), the following expression for the effect bond length is finally proposed:

$$L_{\mathrm{eff}}=\frac{\arctan\sqrt{\frac{s_{f}-s_{1}}{s_{1}}}}{\sqrt{\frac{\tau_{f}^{}}{s_{f}-s_{1}}\left(\frac{1}{t_{c}E_{c}}+\frac{2b_{c}}{t_{s}b_{s}E_{s}}\right)}}+\frac{\mathrm{artanh}\left(\frac{s_{1}-0.0001s_{f}}{s_{1}}\right)}{\sqrt{\frac{\tau_{f}^{}}{s_{1}^{}}\left(\frac{1}{t_{c}E_{c}}+\frac{2b_{c}}{t_{s}b_{s}E_{s}}\right)}}$$

## 6. Validation of the Proposed Model

Equations (66) and (69) were utilized to calculate the bond strength $F_{u}$ and effective bond length $L_{\mathrm{eff}}$, respectively, for CFRP plates externally bonded to corroded steel substrates. Where $b_{c}$, $t_{c}$ and $E_{c}$ are the width, thickness and elastic modulus of the CFRP plate, respectively; $b_{s}$, $t_{s}$ and $E_{s}$ are the width, thickness and elastic modulus of the corroded steel plate, respectively; $t_{s}$ can be calculated by Equation (3); $\tau_{f}$, $s_{1}$, $s_{f}$ and $G_{f}$ are the characteristic parameters of the bond–slip model for CFRP plates externally bonded to corroded steel substrates, which can be obtained from Equations (5), (7), (8) and (9), respectively. The bond strength $F_{u}$ and effective bond length $L_{\mathrm{eff}}$ for the double-lap joints between the CFRP plate and corroded steel plate, which have been experimentally studied by Li et al. [12], were estimated by Equations (66) and (69), respectively, and are listed in Table 2. 

**Figure 6:**
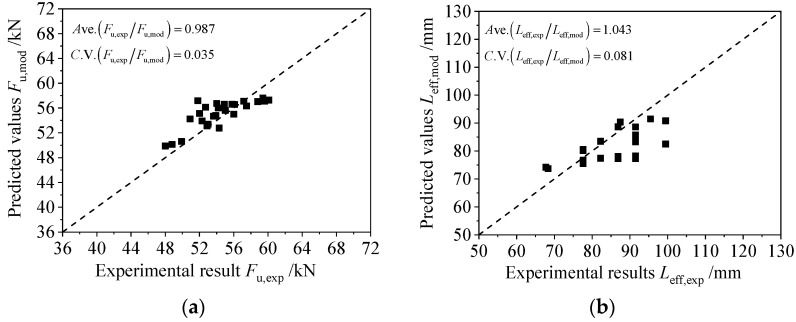
Comparison between predicted values and experimental results of (**a**) bond strength and (**b**) effective bond length.

Figure 6a,b illustrates the typical comparison between the predicted values and experimental results of the bond strength $F_{u}$ and effective bond length $L_{\mathrm{eff}}$, respectively. As Figure 6 shows, it was evident that the predictions were fairly consistent with the experimental results. The ratios between the experimental results and the predicted values for the bond strength had a mean value of 0.987 and a coefficient of variation of 0.035. The ratios between the experimental results and the predicted values for the effective bond length had a mean value of 1.043 and a coefficient of variation of 0.081, indicating that the proposed models of Equations (66) and (69) could be used to estimate the bond strength $F_{u}$ and effective bond length $L_{\mathrm{eff}}$ for the CFRP plates externally bonded to corroded steel substrates with reasonable accuracy. Nevertheless, it is important to note that, due to the aforementioned development of the estimation models being on the basis of the analytical interfacial bond–slip model for a CFRP plate externally bonded to a corroded steel plate of which the characteristic parameters were calibrated according to the fitting results of the test data, the estimation models proposed in this paper, as well as the implicated functions and parameters, are feasible only for the double-lap joints consisting of normal modulus (165 GPa) CFRP plate and linear elasticity adhesive Sikadur-30CN; furthermore, the quantitative rules are restricted to corroded steel plate with the surface preparation method of electric steel wire brush and solvent cleaning. Additional experimental studies and theoretical analysis are still needed to enlarge the application range and improve the accuracy of the models. 

## 7. Conclusions

In the present study, the recent experimental study on the bond behavior between CFRP plate and corroded steel plate was first summarized and then the analytical interfacial bond–slip model for CFRP plate externally bonded to corroded steel plate was proposed. Then, the theoretical stress analysis of the double-lap joint between CFRP plate and corroded steel plate was carried out based on several simplified assumptions; in addition, the piecewise expressions of the interfacial shear stress and the normal peel stress of the interface between the CFRP plate and corroded steel plate were formulated by solving differential equations. Finally, the estimation models of the ultimate load (bond strength) and effective bond length for a double-lap joint between CFRP plate and corroded steel plate were developed on the basis of interfacial stress distribution equations and stress boundary conditions, and the comparison between the predicted values and experimental results indicated that the proposed models could be used to estimate the bond strength and effective bond length for CFRP plates externally bonded to corroded steel substrates with reasonable accuracy. The outcome of this study can provide meaningful references and essential data for the reliable application of a CFRP strengthening system for performance improvement of corroded steel structures.

## Figures and Tables

**Table 1:** Material properties.

Material	Specification	Thickness/mm	Elasticity Modulus/GPa	Yield Strength/MPa	Tensile Strength /MPa	Elongation at Break/%
CFRP plate	CFP-1-514	1.4	165 ^a^	N/A	2400 ^a^	1.61 ^a^
Adhesive	Sikadur-30CN	N/A	5.3	N/A	41.75	1.13
Steel plate	Q235B	10.75 ^b^	181.9	275.6	421.18	20.78

Notes: ^a^ According to the manufacturer’s instructions, ^b^ thickness of steel plate cut from the flange of the uncorroded H beams.

**Table 2:** Comparison between test and predicted values of bond strength and effective bond length.

Specimen No.	*C*_d_/Month	*ξ* /%	*S*_z_/um	*L* /mm	*t*_ave_ /mm	$F_{u,\exp}$ /kN	$F_{u,\mathrm{mod}}$ /kN	$\frac{F_{u,\exp}}{F_{u,\mathrm{mod}}}$	$L_{\mathrm{eff},\exp}$/mm	$L_{\mathrm{eff},\mathrm{mod}}$/mm	$\frac{L_{\mathrm{eff},\exp}}{L_{\mathrm{eff},\mathrm{mod}}}$
C0-B1-T1	0	0.00	157.35	30	0.52	37.00	-	-	-	74.19	-
C0-B2-T1				50	0.47	39.80	-	-	-	73.79	-
C0-B3-T1				80	0.48	48.80	50.12	0.974	-	73.87	-
C0-B4-T1				120	0.52	49.90	50.61	0.986	67.75	74.19	0.913
C0-B5-T1				150	0.46	48.00	49.87	0.963	68.38	73.71	0.928
C0-B5-T2				150	1.19	54.20	56.03	0.967	77.63	80.27	0.967
C0-B5-T3				150	1.51	58.80	57.04	1.031	82.25	83.50	0.985
C0-B5-T4				150	1.72	51.80	57.20	0.906	91.50	85.70	1.068
C3-B5-T1	3	5.08	1301.7	150	0.41	52.00	55.13	0.943	91.50	78.27	1.169
C3-B5-T2				150	0.84	59.60	57.11	1.044	99.50	82.50	1.206
C3-B5-T3				150	1.42	57.18	57.10	1.001	86.88	88.68	0.980
C3-B5-T4				150	1.57	54.90	56.59	0.970	87.50	90.37	0.968
C4-B1-T1	4	6.15	845.95	30	0.45	39.10	-	-	-	76.54	-
C4-B2-T1				50	0.48	43.30	-	-	-	76.81	-
C4-B3-T1				80	0.51	50.90	54.25	0.938	-	77.08	-
C4-B4-T1				120	0.41	53.00	53.39	0.993	-	76.18	-
C4-B5-T1				150	0.47	52.30	53.92	0.970	77.63	76.72	1.012
C6-B5-T1	6	7.92	874.75	150	0.32	54.30	52.77	1.029	77.63	75.52	1.028
C6-B5-T2				150	0.86	55.80	56.62	0.986	77.63	80.56	0.964
C6-B5-T3				150	1.63	60.10	57.28	1.049	91.50	88.64	1.032
C6-B5-T4				150	1.88	57.50	56.34	1.021	95.50	91.49	1.044
C8-B1-T1	8	10.07	937.7	30	1.01	23.28	-	-	-	82.38	-
C8-B2-T1				50	0.36	40.76	-	-	-	76.14	-
C8-B3-T1				80	0.31	52.88	53.11	0.996	-	75.70	-
C8-B4-T1				120	0.49	53.64	54.70	0.981	86.88	77.32	1.124
C8-B5-T1				150	0.50	53.90	54.77	0.984	82.25	77.41	1.062
C8-B5-T2				150	0.79	56.10	56.58	0.992	77.63	80.18	0.968
C8-B5-T3				150	1.10	59.40	57.61	1.031	91.50	83.30	1.098
C8-B5-T4				150	1.79	54.00	56.75	0.952	99.50	90.81	1.096
C12-B1-T1	12	15.02	993.3	30	0.49	40.00	-	-	-	77.58	-
C12-B2-T1				50	0.47	45.60	-	-	-	77.39	-
C12-B3-T1				80	0.62	52.70	56.14	0.939	-	78.80	-
C12-B4-T1				120	0.46	56.00	55.01	1.018	91.50	77.30	1.184
C12-B5-T1				150	0.54	55.00	55.61	0.989	86.88	78.05	1.113

Notes: *C*_d_ is the corrosion duration of the corroded steel plate; *ξ* is weight loss rate of the corroded steel plate; *S*_z_ is the maximum height of the corroded steel surface; *L* is the CFRP bond length of the double-lap joint; *t*_ave_ is the average thickness of both sides of adhesive layers; $F_{u,\exp}$ and $F_{u,\mathrm{mod}}$ are the experimental results and predicted values of bond strength, respectively; $L_{\mathrm{eff},\exp}$ and $L_{\mathrm{eff},\mathrm{mod}}$ are the experimental results and predicted values of effective bond length, respectively.

## Data Availability

All data are shown in the paper.
